# Spatial analysis of the effect of the 2010 heat wave on stroke mortality in Nanjing, China

**DOI:** 10.1038/srep10816

**Published:** 2015-06-02

**Authors:** Kai Chen, Lei Huang, Lian Zhou, Zongwei Ma, Jun Bi, Tiantian Li

**Affiliations:** 1 State Key Laboratory of Pollution Control and Resource Reuse, School of the Environment, Nanjing University, Nanjing, China; 2 Institute for Environmental Health and Related Product Safety, Chinese Centre for Disease Control and Prevention, Beijing, China; 3 Jiangsu Provincial Centre for Disease Prevention and Control, Nanjing, China; 4 School of Government, Nanjing University, Nanjing, China

## Abstract

To examine the spatial variation of stroke mortality risk during heat wave, we collected 418 stroke mortality cases with permanent addresses for a severe heat wave (July 28–August 15, 2010) and 624 cases for the reference period (July 29–August 16, 2009 and July 27–August 14, 2011) in Nanjing, China. Generalized additive models were used to explore the association between location and stroke mortality risk during the heat wave while controlling individual-level risk factors. Heat wave vulnerability was then applied to explain the possible spatial variations of heat-wave-related mortality risk. The overall risk ratio (95% confidence intervals) of stroke mortality due to the heat wave in Nanjing was 1.34 (1.21 to 1.47). Geolocation was found to be significantly associated with the heat-wave-related stroke mortality risk. Using alternative reference periods generated similar results. A district-level risk assessment revealed similar spatial patterns. The highest stroke mortality risk observed in Luhe district was due to the combination of high heat exposure and high vulnerability. Our findings provide evidence that stroke mortality risk is higher in rural areas during heat waves and that these areas require future interventions to reduce vulnerability.

Extreme temperatures have devastating impacts on human health by increasing the mortality and morbidity of the population^1^,^2^,^3^,^4^. Extreme high temperature events, such as the 2003 European heat wave, have attracted increasing attention as global climate change continues^5^. A large number of epidemiology studies have found evidence of an association between heat waves and mortality across various countries^6^,^7^,^8^,^9^,^10^,^11^.

Stroke was the second leading cause of death for 187 countries in 2010^12^. In particular, China has a considerably high stroke mortality, which accounts for 19.9% of the total mortality^13^. Stroke also has an enormous influence on the healthcare expenditures and the Chinese economy, with an annual cost of 40 billion RMB in China^14^. In addition to several traditional risk factors, such as hypertension, diabetes, obesity, smoking, and physical inactivity, extremely high temperatures have been found to be associated with an increased risk of stroke mortality in China^15^. Previous studies have shown that heat waves have a strong adverse effect on stroke mortality, which accounted for 6% to 52% of the excess mortality due to a heat wave^16^. A multi-city case-only analysis of 50 U.S. cities also showed that extremely high temperature days posed a higher relative stroke mortality risk than pneumonia and cardiovascular disease^17^. Therefore, analysing the stroke mortality risk during heat waves is crucial in order to reduce the burden of stroke.

Effective interventions should be implemented to reduce the burden of stroke caused by heat waves. However, previous heat waves risk studies have mainly consisted of population-based ecological analyses^18^,^19^. Even though heat waves could be identified as an important risk factor in these studies, the highest risk area and the underlying causes for this risk remain unknown. Without a spatial distribution of heat-wave-related stroke risk and its risk factors, targeted and enforceable measures cannot be implemented in regions that are in urgent need of intervention.

Spatial analysis can reveal the spatial heterogeneity of heat-wave-related stroke mortality risk and its underlying causes. The spatial heterogeneity of heat wave mortality risk has been demonstrated in several multi-city time series analyses^6^,^8^,^20^. However, most of these studies constitute large-scale regional risk assessments, which aggregated death cases on the level of an entire city. Although spatial analysis has been widely used in the field of environmental epidemiology to investigate the spatial distribution of cancer and respiratory diseases, very limited papers have reported the spatial variations of heat wave effects at a sub-city level^21^. Studies in Sydney and Paris reported that the excess mortality during heat waves presented distinct spatial variations within cities^21^,^22^. We employed a spatial point pattern analysis to explore the risk of heat waves on stroke mortality at a sub-city level. Compared with a regional risk assessment, the spatial analysis of individual cases can (1) investigate the heat wave effect on stroke at a refined spatial level; (2) control the confounding effects of individual level factors; (3) and identify whether geolocation is a risk factor for stroke mortality risk during the heat wave. To analyse potential causes of the spatial heterogeneity of stroke mortality risk during heat waves, we investigated the spatial distribution of the temperature exposure and the heat vulnerability, through which targeted interventions can be determined.

Nanjing, the capital of Jiangsu province in China (Fig. 1), has a large area of 6596 km^2^ and a clear urban-rural gap in socioeconomic levels. In July and August 2010, Nanjing experienced a severe heat wave, which substantially increased the cardiovascular mortality^23^. This heat wave event in Nanjing offered an opportunity to study the spatial heterogeneity of the heat wave effect on stroke mortality at a sub-city level. The objectives of this study were to examine the spatial variation of stroke mortality risk during the heat wave and its causes, as well as to identify high-risk areas and future intervention strategies.

## Results

The average daily maximum temperatures during the study period were 36.1 °C for the heat wave, 29.5 °C for the reference period A2, and 33.3 °C for the reference period A3 (see Supplementary Figure S1 and Table S4). The hottest day of the heat wave occurred on August 13, 2010, with a T_max_ of 38.4 °C.

Our study included 418 stroke mortality cases for the heat wave (A1) and 624 cases for the reference period (A2 and A3) in Nanjing (Table 1). Elderly people, women, unmarried people, people with lower education level, and farmers more frequently suffered from stroke deaths. Approximately 80% of stroke deaths in Nanjing occurred at home, which assured that the stroke death data with permanent addresses were suitable for the spatial analysis. Figure 2 shows the distribution of stroke mortality cases during the 2010 heat wave and reference periods in Nanjing using permanent addresses. Kernel density of stroke death cases was applied in this figure to preserve confidentiality. Higher density of stroke deaths were observed in the north part of Nanjing (Luhe district) in heat wave (A1) compared with reference periods (A2 and A3).

**Figure 1:**
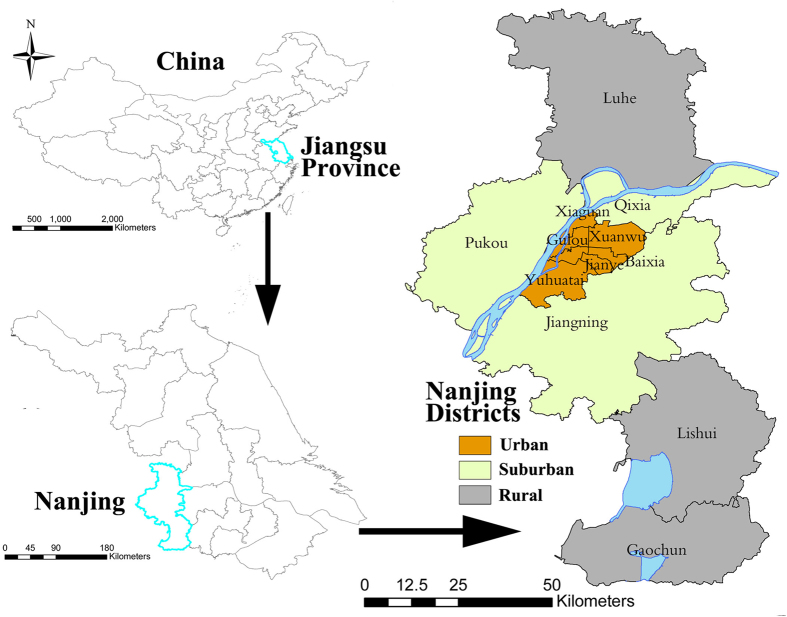
Locations of districts in Nanjing, China. Maps were generated using ArcGIS (version 10.0; ESRI, Redlands, CA).

**Figure 2:**
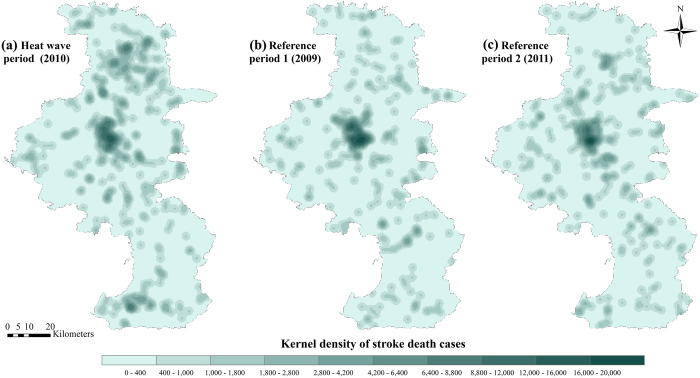
Distribution of stroke mortality cases in A1 (heat wave), A2 (reference period 1) and A3 (reference period 2). Kernel density of cases was used to preserve confidentiality. Maps were generated using ArcGIS (version 10.0; ESRI, Redlands, CA).

**Figure 3:**
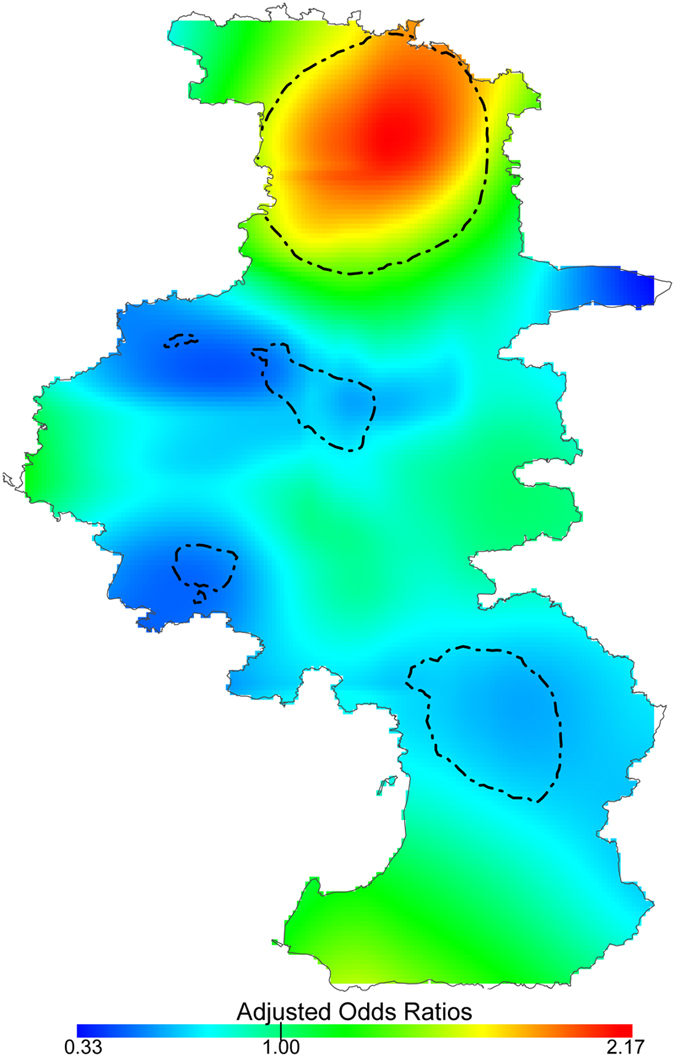
Spatial distribution of Adjusted Odds Ratios (AOR) for total stroke mortality (I60–I64) during the 2010 heat wave in Nanjing. The contour lines show the areas with significantly increased or decreased AOR (p-value < 0.05). This map was generated using R software (version 2.15.0; R Foundation for Statistical Computing, Vienna, Austria).

Based on the deviance statistic of comparing the GAMs with and without spatial locations, the AOR showed significant spatial variations for stroke mortality between the heat wave and the reference period (global p-value < 0.01) (Fig. 3). A significantly increased risk for stroke mortality was observed in the Luhe district (north of Nanjing, a rural district), and significantly decreased risks were observed in urban districts (middle of Nanjing) and the Lishui district (Fig. 1). Different reference periods yielded similar ranges in AOR (using A2: 0.45 to 2.19; using A3: 0.55 to 2.55) (see Supplementary Figure S2).

The 2010 heat wave significantly increased the stroke mortality in Nanjing (RR = 1.34, 95% CI: 1.21 to 1.47) (Table 2). For rural area in Nanjing, the RR was 1.89 (95% CI: 1.63 to 2.17), which indicated significantly increased risks of stroke mortality in rural areas. However, we did not find significant risks in urban districts. In suburban districts, the RRs were slightly higher than 1 but not statistically significant. The heat wave risk did not significantly differ between alternative reference periods (see Supplementary Table S2).

**Figure 4:**
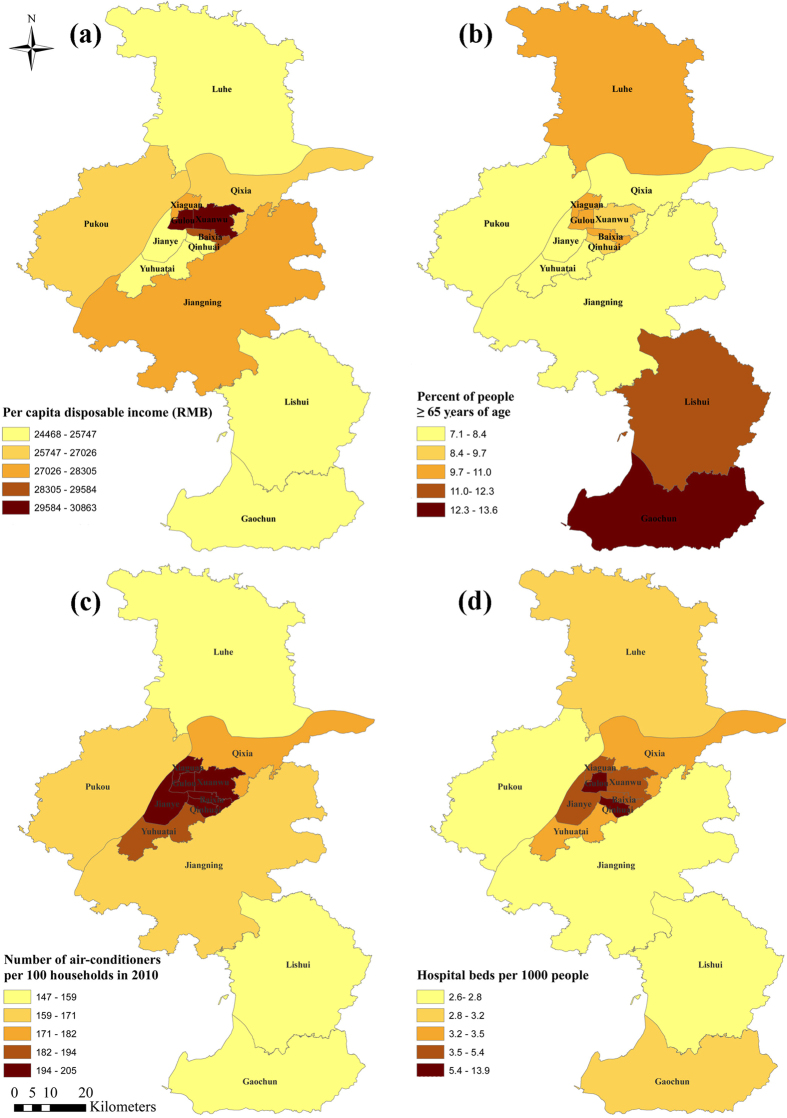
Heat wave vulnerability factors in different districts of Nanjing: (**a**) Per capita disposable income (RMB); (**b**) Percent of people ≥65 years old; (**c**) Number of air-conditioners per 100 households in 2010; (**d**) Hospital beds per 1000 people. Maps were generated using ArcGIS (version 10.0; ESRI, Redlands, CA).

The heat-related vulnerability varied among different districts in Nanjing (Fig. 4). Compared with urban districts, the socioeconomic level (pdincome) and prevalence of air conditioning (AC) of rural districts (Luhe, Lishui, and Gaochun) was low, while the percentage of residents aged ≥65 years (pelderly) was high and the level of health care (pHbed) was insufficient for this population.

**Figure 5:**
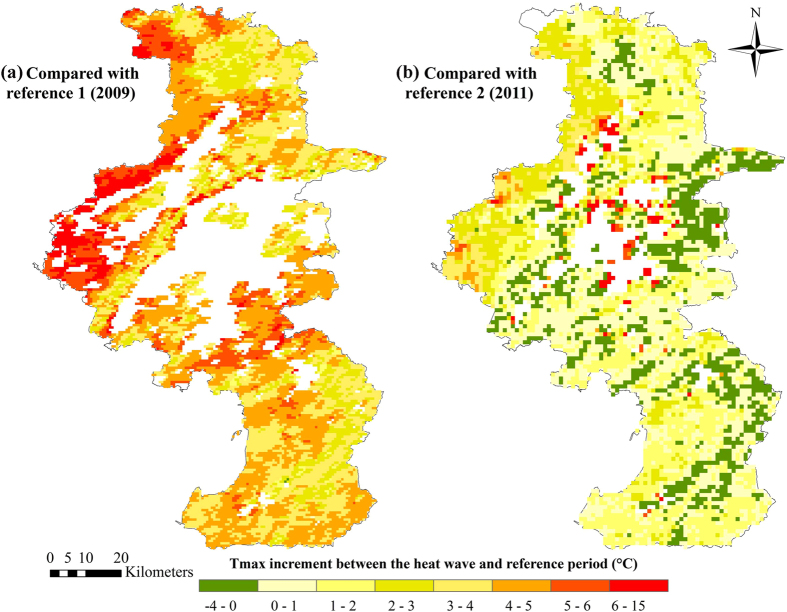
Maximum temperature exposure during the heat wave compared with different reference periods. (1) Using reference period 1 (A2); (2) Using reference period 2 (A3). Maximum of daytime land surface temperatures (Terra/MODIS, 1 km resolution) in each period (19 days) was used as the temperature exposure indicator. White areas indicate that land surface temperatures were not available due to cloud cover. Maps were generated using ArcGIS (version 10.0; ESRI, Redlands, CA).

The maximum LST in urban districts was not available due to cloud cover (Fig. 5). The increase in the maximum LST between the heat wave and reference period were generally much higher in the Luhe district than in the Lishui or Gaochun district. Due to the lower temperatures in reference A2 (see Supplementary Figure S1), the increase in the maximum LST between the heat wave and reference period A2 was higher than that between the heat wave and reference period A3.

## Discussion

Compared with the reference period in 2009 and 2011, the risk for stroke mortality in Nanjing was high during the 2010 heat wave. The spatial analysis revealed significant spatial variations in the stroke mortality risk between the heat wave and the reference period. Rural districts, especially the Luhe district, had significantly higher stroke mortality risks than urban districts during the heat wave. To our knowledge, this study contains the first spatial analysis to examine the within-city spatial variation of heat-wave-related stroke mortality risk in China.

We found a significantly high risk of stroke mortality during the heat wave (RR = 1.34, 95% CI: 1.21 to 1.47) in Nanjing (Table 2). This result is consistent with another episode analysis in Shanghai, which is near Nanjing. The risk of the 2003 heat wave in Shanghai was 1.21 (95% CI: 1.06 to 1.39) for stroke mortality, which was as high if not greater than that for total (RR = 1.13, 95% CI: 1.06 to 1.20) and cardiovascular (RR = 1.19, 95% CI: 1.08 to 1.32) mortality^9^.

Another major finding of this study is that geolocation was a significant risk factor for stroke mortality during the heat wave in Nanjing. Significantly increased risks were observed in the Luhe district, while decreased risks were found in urban districts and the Lishui district (Fig. 3). The district-level risk estimation revealed similar spatial patterns of stroke mortality risks, see Supplementary Table S1 and Supplementary Figure S3. The significantly decreased risks areas revealed by the spatial point pattern analysis were not statistically significant in the district-level risk estimation (see Supplementary Table S1). This inconsistency may be driven by the small number of stroke cases in these areas (<100 cases during the heat wave). Thus, the significantly decreased risk areas found in the spatial point pattern analysis may be due to contingency. On the contrary, the consistent results obtained from different analysis methods and relatively large number of stroke cases verified the significantly increased risk area in the Luhe district.

Compared with urban areas, the risk for stroke mortality during the heat wave was significantly higher in rural areas according to this analysis (Fig. 3). Few studies have examined the health effect of heat waves on mortality at a sub-city level, and the results remain controversial. Consistent with this study, a county-level analysis of heat vulnerability across Ohio showed the percentage of mortality increased more markedly in rural and suburban counties than in urban counties^24^. On the contrary, the total mortality associated with the 1980 heat wave in Missouri increased by 57% in St Louis City and 64% in Kansas City, but only 10% in the rural areas of Missouri^25^. Moreover, other western studies showed that people living in cities and rural areas may be equally vulnerable to heat-related mortality risks. During the heat waves that occurred between 1990 and 2006, similar mortality risks were observed in the city of Berlin and the rural Federal State of Brandenburg, though the mortality risks were higher in urban areas than in rural areas during the two main heat waves within the study period^26^. Another study that evaluated the heat-related vulnerability in England and Wales also demonstrated that the risk estimates between urban and rural populations did not significantly differ^27^.

The differences in risk estimates between urban and rural areas may be due to variability in the heat vulnerability in different regions^28^. Because the AOR in Fig. 3 was already adjusted for individual risk factors, the observed spatial variation may be due to the spatial heterogeneity of regional heat-related vulnerability. Elderly people have been proven to be extremely vulnerable to heat-related mortality^7^,^27^,^29^ and have a higher mortality during heat waves^8^,^10^,^18^. Income-related variability is also an important risk factor of heat-related mortality^6^,^18^. As a strong adaptation to high temperatures, air conditioning has been demonstrated as a protective factor against the heat-related mortality^30^. The number of hospital beds per capita was used here as an indicator of medical service^31^. As expected, pdincome, AC, and pHbed were negatively associated with district-level stroke mortality risks, while pelderly showed a positive association (see Supplementary Figure S4). Rural districts were associated with low personal disposal income, high percentages of elderly people, low prevalence of AC, and insufficient hospital resources, which made them more vulnerable than urban districts during the 2010 heat wave in Nanjing (Fig. 4).

Compared with other rural districts (Lishui and Gaochun), the Luhe district was exposed to much higher heat (Fig. 5). Thus, the significantly increased stroke mortality risk in the Luhe district can be inferred to be due to the high vulnerability and high exposure. For the other rural districts, the maximum LST during the heat wave was not much higher than those during the two reference periods. The low heat exposure in the Gaochun district may result in a lower heat wave risk compared with the Luhe district (Fig. 3). Further studies are needed to quantify the contribution of heat exposure and vulnerability characteristics with observed heat-wave-related risks.

Our findings contradict the general assumption that people living in urban areas are more vulnerable than those living in rural areas^26^. Because of the urban heat island effect, urban residents are thought to be more vulnerable to hot weather^32^. However, this assumption might ignore the fact that the health impact of a hazard event is determined not only by exposure but also by the population vulnerability^33^. The urban heat island effect clearly increases the heat exposure of urban residents. Therefore, the decreased stroke mortality risk in urban areas is mainly due to the low vulnerability found in those areas (Fig. 4).

The mortality rate due to stroke is higher in urban areas than in rural areas in China^14^. However, our findings indicate that the heat-wave-related stroke mortality risk is higher in rural areas than in urban areas. This inconsistency implies that stroke prevention and health care may be neglected in rural areas during the heat wave. Compared with urban citizens, the use of CT scanning in patients with stroke and the percentage of the population with health insurance were much lower in rural areas^34^. Stroke is an emergency and needs immediate treatment^14^. Reducing the population vulnerability can prevent heat-stroke-related mortality. Identifying areas in which the heat-related stroke mortality is high and the underlying causes for this high mortality can help design focused interventions to reduce the burden of stroke during heat waves. Thus, more emphasis should be placed on the fair distribution of heat-related vulnerability factors within Chinese cities to respond to the challenges that heat waves will pose during climate change. We found that the stroke mortality risk was significantly higher in the Luhe district during the heat wave, and future interventions should focus on this district. Targeted intervention strategies should include measures to reduce heat-related vulnerability, such as the prevalence of AC use and distribution of medical resources.

Our analysis has several strengths. First, stroke death in Nanjing mainly occurred at home, which offered a good opportunity to examine the spatial variation of heat-wave-related mortality risks. Furthermore, both individual risk factors and regional vulnerability factors were taken into consideration, which allowed the spatial heterogeneity of heat-wave-related stroke morality risks to be examined. In addition, different reference periods were used for a sensitivity analysis, which made our results more plausible.

Our study features several limitations. First, the limited amount of stroke mortality data in our study periods may lead to unstable results. Future studies should collect data from additional cities in order to ensure sufficient statistical power to investigate the spatial variation of stroke mortality risks during heat waves. Second, we were unable to adjust some individual risk factors for stroke mortality, such as smoking^35^. Although smoking prevalence was not available in the death certificates in Nanjing, its effect on stroke mortality might be much smaller than the large heat wave effect in such a short period (19 days). Moreover, the smoking prevalence in China is closely related to age, gender, education, and occupation^36^. The difference in the smoking prevalence between heat wave stroke cases and the reference period stroke cases might be reflected by these already controlled individual covariates to some extent. Third, this study did not adjust for air pollutants because the rural areas in Nanjing lacked monitoring stations. However, this lack of data may not be a major issue because a previous study indicates that air pollutants have little influence on the heat-related stroke mortality risk^15^.

In conclusion, we found that the stroke mortality risk due to heat waves in Nanjing had clear spatial patterns. During the heat wave, the stroke mortality risk was highest in the Luhe district. Vulnerability played an important role in the impact of heat waves on health at a sub-city level. This study added evidence to the spatial variability of heat wave-related mortality risk at a sub-city level, which strengthened the need of local level vulnerability pattern analysis. These findings also suggested that climate change-related mortality risk was not limited to urban areas and revealed the importance of heat wave adaptation planning strategies in rural areas. Future heat wave studies with a high spatial resolution similar to this one, could provide better understanding of the spatial variation of heat-wave-related mortality risk and more precise information on heat-related deaths prevention.

## Methods

Our study area included all 13 districts of Nanjing, 3 of which are rural districts (Fig. 1), which contained a population of 8.0 million by the end of 2010.

### Data collection

The Jiangsu Provincial Centre for Disease Prevention and Control provided the daily mortality data with permanent addresses. The stroke mortality counts were calculated using the International Classification of Diseases, Revision 10 (ICD-10): total stroke (codes I60–I64) for all ages. Stroke deaths of non-Nanjing residents were excluded. Based on the permanent addresses and online maps (Google maps and Baidu maps), we geocoded all stroke mortality cases using ArcGIS. Individual heat-related risk factors, such as age, gender, and education attainment, were also included in the mortality data. Nearly all stroke deaths (99.5%) were in Han Chinese individuals; thus, racial disparity was not an issue in this study. The daily maximum temperatures (T_max_) were obtained from the China Meteorological Data Sharing Service System.

To explain the spatial variation of heat wave vulnerability, we chose four variables that have been demonstrated in the literature to increase the regional vulnerability of heat-related mortality: (1) per capital disposal income (pdincome), which represents the regional economic level; (2) the percent of people ≥65 years of age (pelderly), which served as the demographic variable; (3) the number of air conditioning units per 100 households (AC), which represented the cooling capacities of indoor environments; (4) and the number of hospital beds per 1000 people (pHbed), which demonstrates the health care level^31^,^37^,^38^,^39^. The pelderly data were obtained from the tabulation of the 2010 Population Census of China. The other three variables (pdincome, pHbed, and AC) for the year 2010 were collected from the Nanjing Statistics Yearbook. All of these variables were at the district-level within Nanjing.

We calculated the increase in the maximum land surface temperature (LST) between the heat wave and reference period (defined below) to explain the spatial variation of heat wave exposure. Daily MODIS collection of 5 LST at 1 km (MOD11A1 from TERRA) were provided by International Scientific & Technical Data Mirror Site, Computer Network Information Centre, Chinese Academy of Sciences (http://www.gscloud.cn). The maximum LST for pixels during the heat wave and reference periods were calculated using the fuzzy maximum overlay function in ArcGIS.

### Heat wave and reference period definition

Heat waves lack a universal definition, and different definitions may influence the estimates of the heat wave health effects^40^. Because we aimed to study the spatial variation of heat wave health effects instead of the absolute estimates, we used the stricter heat wave definition of Meehl and Tebaldi^5^ by calculating the 97.5^th^ percentile of the distribution of the daily maximum temperatures (Threshold 1) and the 81^st^ percentile of the daily maximum temperatures (Threshold 2). In this study, a heat wave was defined as the longest period of consecutive days that satisfy the following conditions: (1) T_max_ is above Threshold 1 for at least 3 days, (2) the average T_max_ for the entire period is above Threshold 1, (3) T_max_ is above the Threshold 2 for every day of the period^5^. Thus, a heat wave period, A1 (July 28–August 15, 2010), was defined by analysing the T_max_ in Nanjing from 2000–2012.

To calculate the risk of a heat wave, we defined the reference period as non-heat wave days of the same calendar period in adjacent years. The same calendar period between the heat wave and reference period was chosen to control effects of the day of the week and potential time-varying confounding effects. Based on this definition, the reference period (July 29–August 16, 2009 and July 27–August 14, 2011) was selected. Besides, two separate reference periods, A2 (July 29–August 16, 2009) and A3 (July 27–August 14, 2011), were used in the spatial analysis to evaluate the sensitivity to the selection of the reference period.

### Spatial heat waves risk analysis

In this study, an episode analysis was conducted by comparing the mortality between heat wave and non-heat wave periods in order to investigate the overall health effect of heat stress during the heat wave. With an average annual growth rate of 2.5%, the population of Nanjing changed little from 2009 to 2011. This relatively stable study population and the same calendar period allowed us to compare the number of cases in the heat wave and that in the reference period to indicate the relative health effect of heat waves^9^.

A spatial point pattern analysis was conducted to explore whether location was a significant risk factor associated with the stroke mortality risk during the heat wave. In spatial point pattern analysis, binary regressions that use generalized additive models have the advantages of allowing the inclusion of covariates in the model by means of logistic regression and exploring the spatial variation of risks via a smooth spatial function^41^. Compared with non-heat wave periods, the risks for stroke mortality by location during the heat wave were estimated by using generalized additive models (GAMs) with LOESS smoothing for geolocations (paired x and y coordinates):
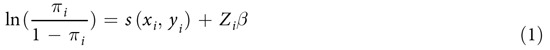
where *π*_*i*_ is the probability for stroke mortality during the heat wave for case *i*, ln(*π*_*i*_/(1 −*π*_*i*_)) is the log odds of the stroke mortality probability during the heat wave to that during the reference period for case *i*, *x*_*i*_ and *y*_*i*_ are the spatial coordinates of case *i*, *s(.,.)* is the LOESS function, and *Z*_*i*_ is the individual covariate vector. By minimizing the Akaike Information Criterion, the optimal span sizes used for the LOESS smooth for location were determined to be 0.3. Because the default convergence criteria in the GAM may overestimate the effect estimates and underestimate the standard errors, we used a more stringently convergence criteria (i.e., 1e-15 for the convergence precision and 1000 for the maximum number of iterations ) suggested by Dominici *et al.*^42^.

Adjusted odds ratios (AOR) were applied in this study to represent the spatial variation of the stroke mortality risk after adjusting for individual-level risk factors^43^. The estimated AOR for each point on the grid was calculated as the ratio between the log odds from the spatially smoothed GAMs and that from the same logistic model without geolocation. A permutation test was conducted 999 times to measure the significance of the smoothed geolocation. For each permutation test, the geolocations of stroke mortality cases were randomly reassigned to the heat wave period and the reference period. This process is consistent with the null hypothesis, which states that geolocation is not associated with the stroke mortality risk. For each point, the percentile rank of the local log odds ratio were calculated by comparing them to the local log odds ratio distribution from the permutation test. Spatial regions with statistically significant AOR were mapped using these point-wise permutation percentile ranks.

The following covariates were adjusted for confounding in the GAMs: age (modelled as a continuous variable), gender (male or female), marriage (married or not), and occupation. Occupation was grouped into 4 categories: agriculture, household, unemployed, and other occupations. Covariates were included in the GAMs if they were risk factors of stroke mortality and were associated with heat wave exposure. Wilcoxon–Mann–Whitney tests between heat wave period (A1) and the reference period (A2 or A3) showed that there were significant differences for gender, marriage, and occupation (see Supplementary Table S3). Since age is generally a critical adjustment factor for a mortality study, it was also included in the GAMs.

We also calculated the risk ratio (RR) for each district and the entire area of Nanjing as the ratio of the stroke death cases during the heat wave period to those in the reference period. This district-level RR was applied to estimate the overall stroke mortality risk during the heat wave and to verify the spatial pattern found in the individual-level spatial data analysis. The 95% confidence intervals (95% CIs) for RR were calculated using the pois.exact function in the epitools package in R. Spatial distribution of heat vulnerability factors were then used to illustrate the difference in heat vulnerability between urban and rural areas.

All analyses were conducted in ArcGIS (version 10.0; ESRI, Redlands, CA) and R 2.15.0 using the MapGAM package (version 0.6–2; R Foundation for Statistical Computing, Vienna, Austria).

## Additional Information

**How to cite this article**: Chen, K. *et al.* Spatial analysis of the effect of the 2010 heat wave on stroke mortality in Nanjing, China. *Sci. Rep.* **5**, 10816; doi: 10.1038/srep10816 (2015).

## Supplementary Material

Supplementary Information

## Figures and Tables

**Table 1:** Summary of characteristics (number and %) of stroke mortality cases in heat wave period (A1) and reference period (A2 and A3).

**Characteristics**	**A1, n (%)**	**A2, n (%)**	**A3, n (%)**
Number of cases	418(100.0%)	312(100.0%)	312(100.0%)
Age (years)			
<45	6(1.4%)	6(1.9%)	3(0.9%)
45–54	20(4.8%)	13(4.2%)	13(4.2%)
55–69	75(18.0%)	60(19.2%)	59(18.9%)
≥70	317(75.8%)	233(74.7%)	237(76.0%)
Gender			
male	184(44.0%)	139(44.6%)	171(54.8%)
female	234(56.0%)	173(55.4%)	141(45.2%)
Marital status			
married	250(59.8%)	212(67.9%)	191(61.2%)
others	168(40.2%)	100(32.1%)	121(38.8%)
Education			
illiterate or primary	344(82.3%)	240(76.9%)	240(76.9%)
high school or college	55(13.2%)	60(19.2%)	54(17.3%)
others	19(4.5%)	12(3.9%)	18(5.8%)
Occupation			
agriculture	225(53.8%)	141(45.2%)	158(50.6%)
household	57(13.6%)	40(12.8%)	32(10.3%)
unemployed	63(15.1%)	40(12.8%)	41(13.1%)
others	73(17.5%)	91(29.2%)	81(26.0%)
Death location			
home	348(83.3%)	248(79.5%)	251(80.4%)
hospital	59(14.1%)	61(19.6%)	60(19.2%)
others	11(2.6%)	3(1.0%)	1(0.3%)

**Table 2:** Risk ratio (95% CI) of the 2010 heat wave on stroke mortality in Nanjing.

**Region**	**Heat wave cases**	**Reference period cases**^a^	**RR**^b^
Urban districts	96	102	0.94(0.76,1.15)
Suburban districts	125	105.5	1.18(0.99,1.41)
Rural districts	197	104.5	1.89(1.63,2.17)
Nanjing	418	312	1.34(1.21,1.47)

^a^Using average cases of A2 period and A3 period.

^b^RR was calculated as the ratio between stroke deaths in the heat wave and in the reference period.
