# Supplementary material for: Spatial analysis of the effect of the 2010 heat wave on stroke mortality in Nanjing, China
Source: Sci Rep. 2015 Jun 2;5:10816. doi: 10.1038/srep10816 (PMC4451699; doi:10.1038/srep10816)
Supplement: Supplementary Information [file srep10816-s1.pdf]

Supplementary Information

Spatial analysis of the effect of the 2010 heat wave on stroke mortality in Nanjing, China

Kai Chen, Lei Huang, Lian Zhou, Zongwei Ma, Jun Bi and Tiantian Li

Table of Contents Page

Tables

Supplementary Table S1. Risk ratios of heat wave on stroke mortality in different districts of Nanjing City. 4

Supplementary Table S2. Risk ratios (95% CI) of heat wave on stroke mortality in Nanjing using different reference periods. 5

Supplementary Table S3. Significance of differences (p-values) in characteristics of stroke mortality cases between heat wave period (A1) and the reference period (A2 and A3) using the Wilcoxon–Mann–Whitney test. 6

Supplementary Table S4. Summary of average daily maximum temperature (°C) and number of stroke mortality cases by urbanity in heat wave period (A1) and reference period (A2 and A3). 7

Figures

Supplementary Figure S1. Daily maximum temperature during the 2010 heat wave (A1) and the reference period (A2 and A3). 8

Supplementary Figure S2. Spatial distribution of Adjusted Odds Ratios (AOR) for total stroke mortality (I60-I64) using different reference periods. (a) Using A2 stroke as reference; (b) Using A3 stroke as reference. The contour lines show the areas with significantly increased or decreased AOR (p-value < 0.05). Maps were generated using R software (version 2.15.0; R Foundation for Statistical Computing, Vienna, Austria). 9

Supplementary Figure S3. Relative risks of heat wave on stroke mortality in different districts of Nanjing. This map was generated using ArcGIS (version 10.0; ESRI, Redlands, CA). 10

Supplementary Figure S4. Scatter plot of heat wave risks and vulnerability factors in different districts of Nanjing. RRs stands for relative risks, pdincome is the per capita disposable income (RMB), pelder is the percent of people ≥ 65 years of age, AC is the number of air conditioning units per 100 households in 2010, and pHbed is the hospital beds per 1000 people. Linear regression lines are also plotted. 11

Supplementary Figure S5. Spatial distribution of Adjusted Odds Ratios (AOR) for total stroke mortality (I60-I64) using different modeling choices. (a) Using the default convergence criteria and controlling gender, marriage, and occupation; (b) Using a more stringently convergence criteria and controlling gender, marriage, and occupation; (c) Using the default convergence criteria and controlling individual age, gender, marriage, and occupation; (d) Using a more stringently convergence criteria and controlling individual age, gender, marriage, and occupation. The contour lines show the areas with significantly increased or decreased AOR (p-value < 0.05). Maps were generated using R software (version 2.15.0; R Foundation for Statistical Computing, Vienna, Austria). 12

Supplementary Table S1. Risk ratios (95% CI) of heat wave on stroke mortality in different districts of Nanjing City.

Districts	Heat wave cases	Reference period cases [a]	RR
**Luhe**	121	49.5	**2.44(2.03,2.92)**
**Xuanwu**	5	13	**0.38(0.12,0.90)**
**Baixia**	22	19	1.16(0.73,1.75)
**Qinhuai**	8	15	0.53(0.23,1.05)
**Jianye**	5	9.5	0.53(0.17,1.23)
**Gulou**	21	19.5	1.08(0.67,1.65)
**Xiaguan**	16	13	1.23(0.70,2.00)
**Yuhuatai**	19	13	1.46(0.88,2.28)
**Pukou**	34	33	1.03(0.71,1.44)
**Qixia**	21	16.5	1.27(0.79,1.95)
**Jiangning**	70	56	1.25(0.97,1.58)
**Lishui**	25	29.5	0.85(0.55,1.25)
**Gaochun**	51	25.5	**2.00(1.49,2.63)**
**Urban districts**	96	102	0.94(0.76,1.15)
**Suburban districts**	125	105.5	1.18(0.99,1.41)
**Rural districts**	197	104.5	**1.89(1.63,2.17)**
**Whole Area**	418	312	**1.34(1.21,1.47)**

[a] Using average cases of A2 period (July 29-August 16, 2009) and A3 period (July 27-August 14, 2011).

Supplementary Table S2. Risk ratios (95% CI) of heat wave on stroke mortality in Nanjing using different reference periods [a].

Region	RR [b]	RR [c]
Urban districts	0.82(0.66,1.01)	1.10(0.89,1.35)
Suburban districts	**1.29(1.07,1.54)**	1.10(0.91,1.31)
Rural districts	**2.01(1.74,2.31)**	**1.77(1.54,2.04)**
Nanjing	**1.34(1.21,1.47)**	**1.34(1.21,1.47)**

[a] RR was calculated as the ratio between stroke deaths in the heat wave and in the reference period;
[b] Using A2 period (July 29-August 16, 2009) alone as the reference period;
[c] Using A3 period (July 27-August 14, 2011) alone as the reference period.

Supplementary Table S3. Significance of differences (p-values) in characteristics of stroke mortality cases between heat wave period (A1) and the reference period (A2 and A3) using the Wilcoxon–Mann–Whitney test.

Characteristics	A1 vs. A2	A1 vs. A3
Age	0.299	0.785
Gender	0.886	**0.004**
Marital status	**0.024**	0.700
Education	0.096	0.076
Occupation	**0.002**	0.086
Death location	0.235	0.400

Supplementary Table S4. Summary of average daily maximum temperature (°C) and number of stroke mortality cases by urbanity in heat wave period (A1) and reference period (A2 and A3).

Characteristics	A1, n (%)	A2, n (%)	A3, n (%)
Maximum temperature (°C)	36.12	29.52	33.32
Number of cases	418(100.0%)	312(100.0%)	312(100.0%)
urban	96(23.0%)	117(37.5%)	87(27.9%)
suburban	125(29.9%)	97(31.1%)	114(36.5%)
rural	197(47.1%)	98(31.4%)	111(35.6%)

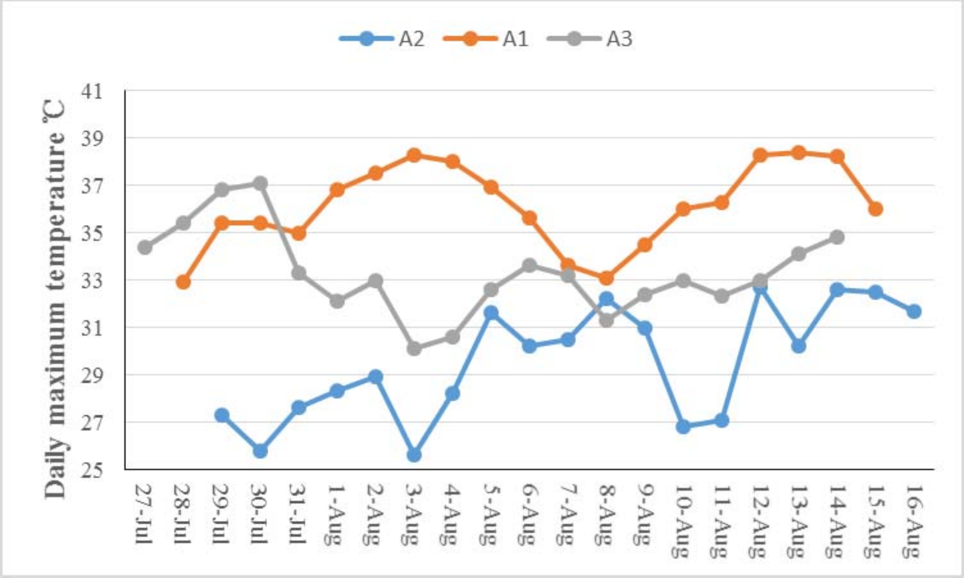

Supplementary Figure S1. Daily maximum temperature during the 2010 heat wave (A1) and two reference periods (A2 and A3).

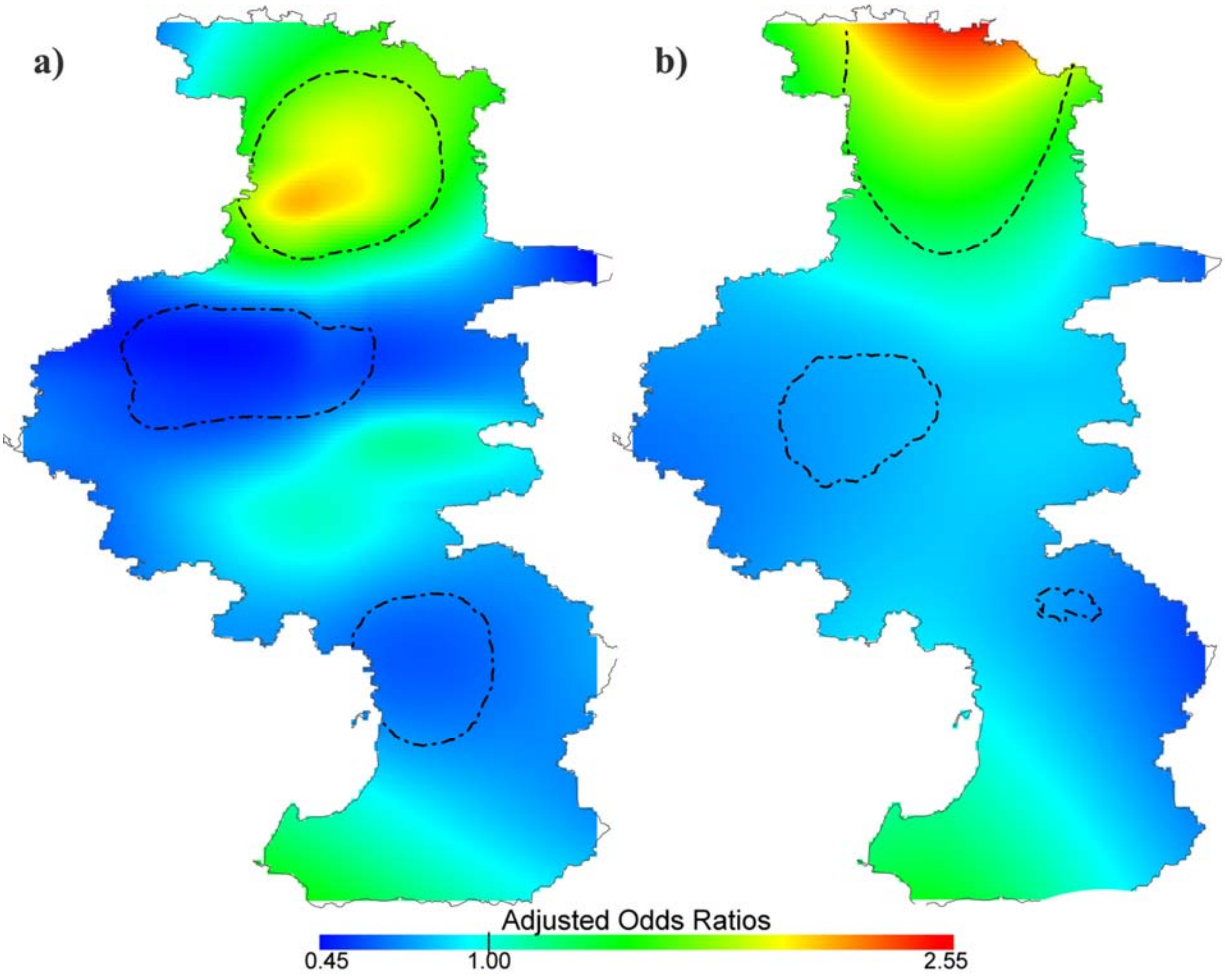

Supplementary Figure S2. Spatial distribution of Adjusted Odds Ratios (AOR) for total stroke mortality (I60-I64) using different reference periods. (a) Using A2 stroke as reference; (b) Using A3 stroke as reference. The contour lines show the areas with significantly increased or decreased AOR (p-value < 0.05). Maps were generated using R software (version 2.15.0; R Foundation for Statistical Computing, Vienna, Austria).

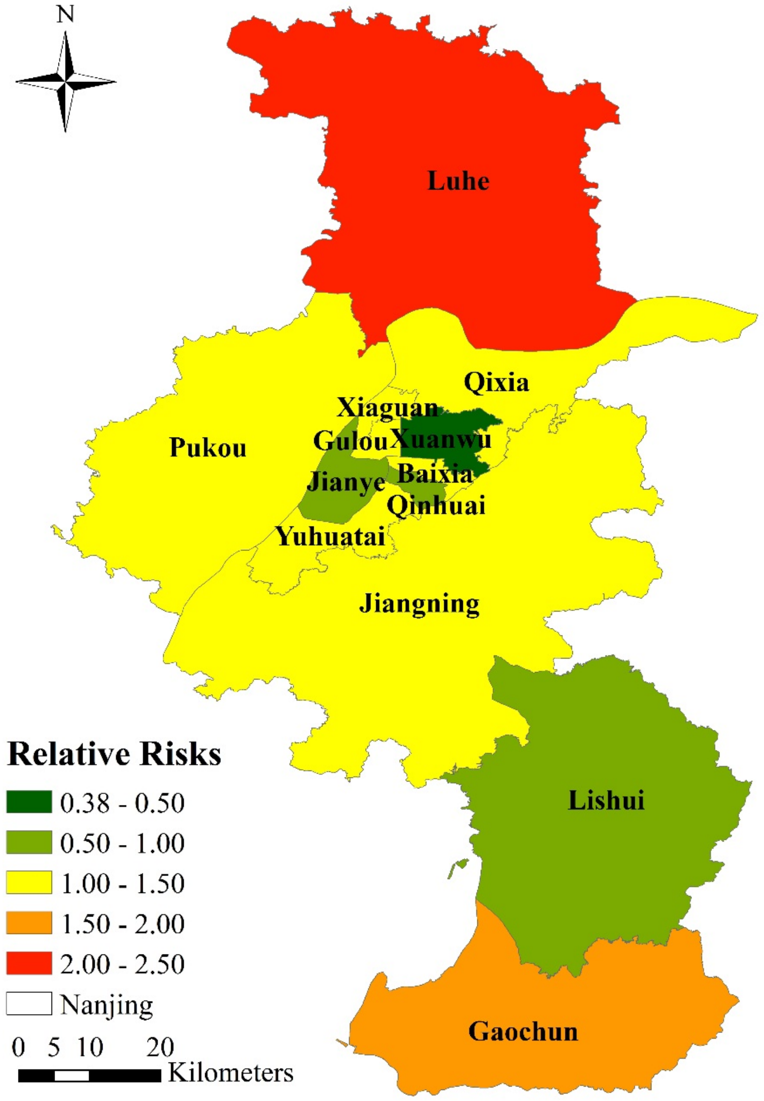

Supplementary Figure S3. Relative risks of heat wave on stroke mortality in different districts of Nanjing City. This map was generated using ArcGIS (version 10.0; ESRI, Redlands, CA).

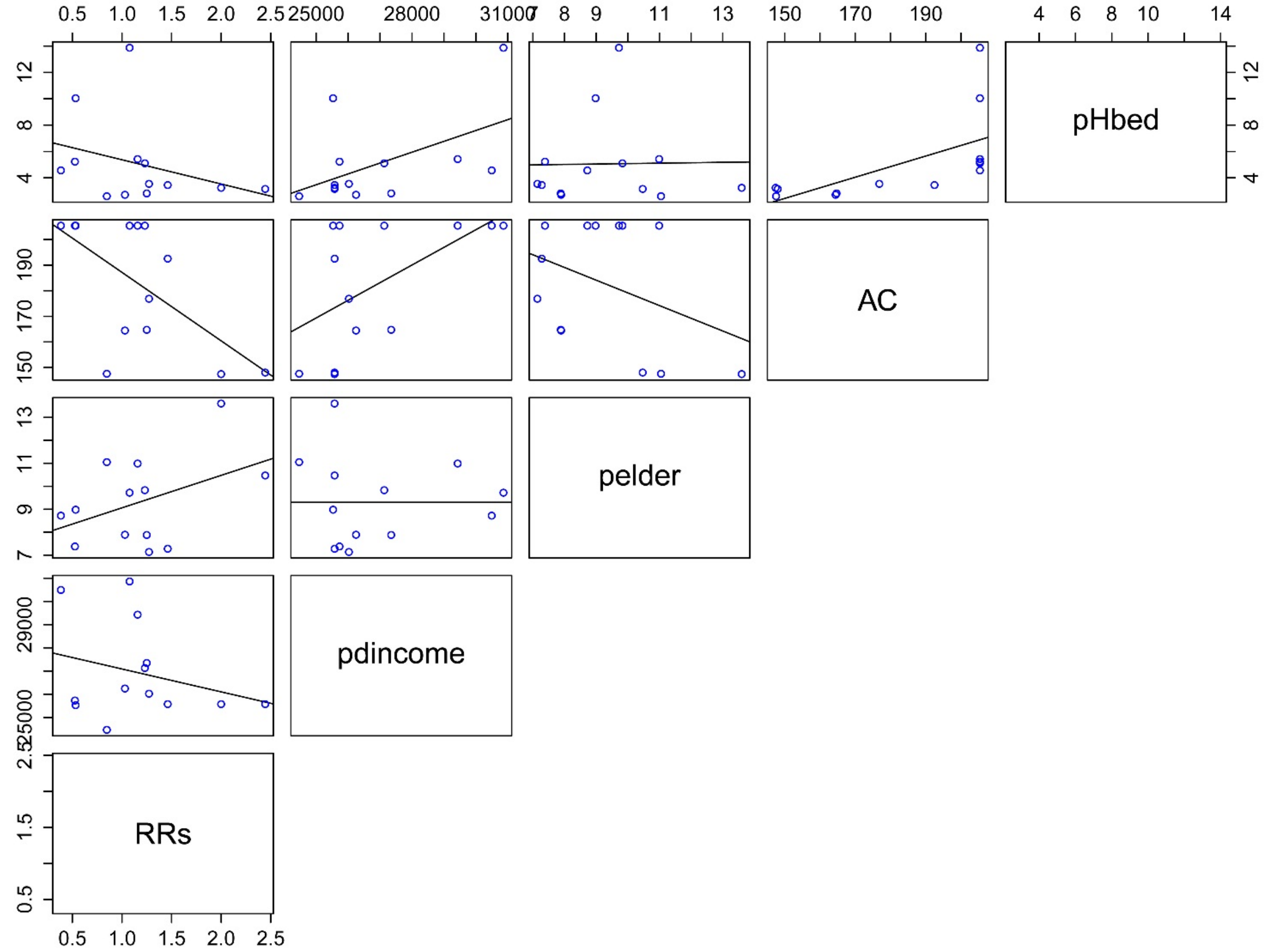

Supplementary Figure S4. Scatter plot of heat wave risks and vulnerability factors in different districts of Nanjing. RRs stands for relative risks, pdincome is the per capita disposable income (RMB), pelder is the percent of people ≥ 65 years of age, AC is the number of air-conditioners per 100 households in 2010, and pHbed is the hospital beds per 1000 people. Linear regression lines are also plotted.

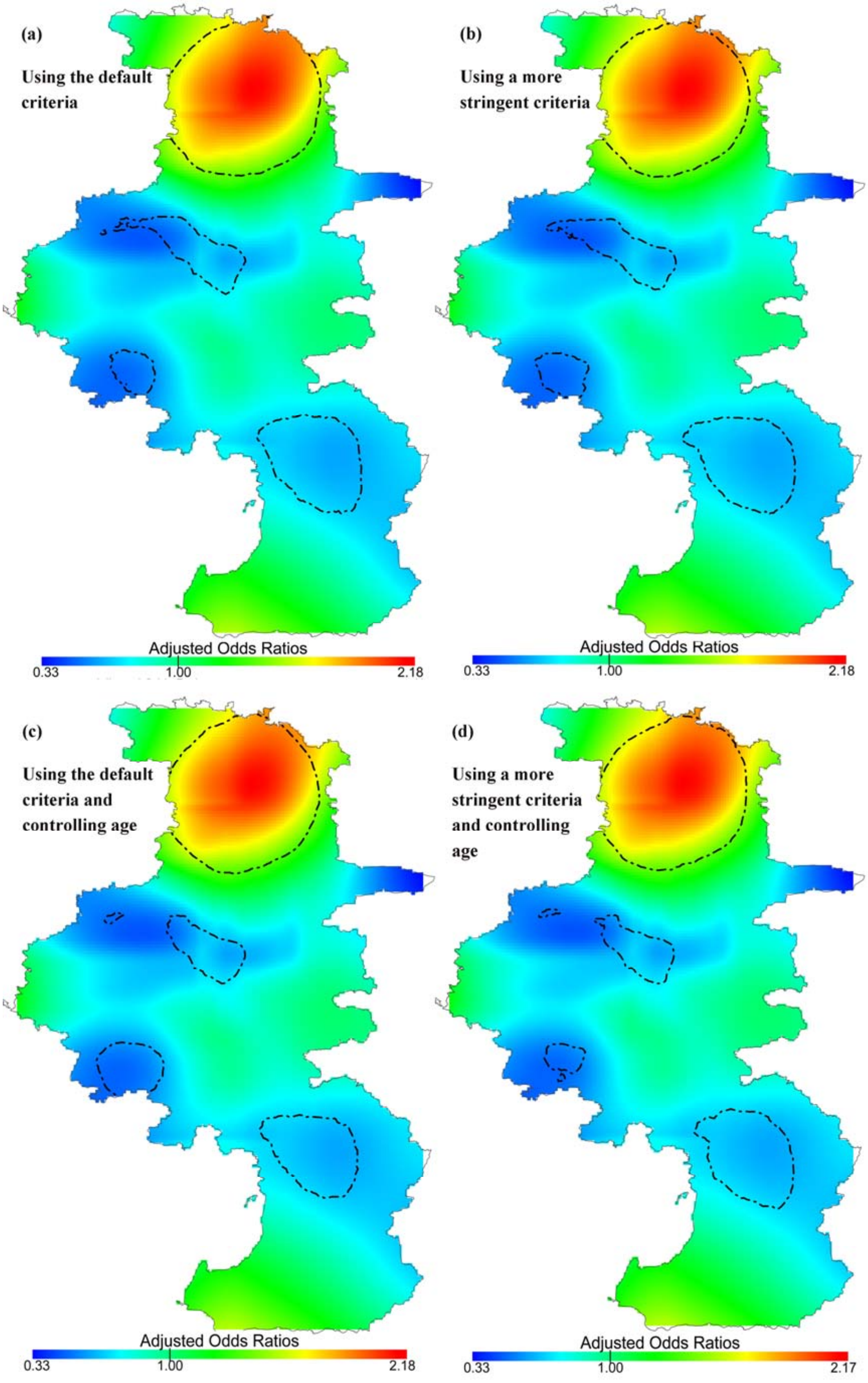
(a)
Using the default criteria
Adjusted Odds Ratios
0.33
1.00
2.18
(b)
Using a more stringent criteria
Adjusted Odds Ratios
0.33
1.00
2.18
(c)
Using the default criteria and controlling age
Adjusted Odds Ratios
0.33
1.00
2.18
(d)
Using a more stringent criteria and controlling age
Adjusted Odds Ratios
0.33
1.00
2.17

Supplementary Figure S5. Spatial distribution of Adjusted Odds Ratios (AOR) for total stroke mortality (I60-I64) using different modeling choices. (a) Using the default convergence criteria and controlling gender, marriage, and occupation; (b) Using a more stringently convergence criteria and controlling gender, marriage, and occupation; (c) Using the default convergence criteria and controlling individual age, gender, marriage, and occupation; (d) Using a more stringently convergence criteria and controlling individual age, gender, marriage, and occupation. The contour lines show the areas with significantly increased or decreased AOR (p-value < 0.05). Maps were generated using R software (version 2.15.0; R Foundation for Statistical Computing, Vienna, Austria).
